# Real-World Functioning in Patients With Schizophrenia: Beyond Negative and Cognitive Symptoms

**DOI:** 10.3389/fpsyt.2021.700747

**Published:** 2021-08-09

**Authors:** María Paz García-Portilla, Leticia García-Álvarez, Leticia González-Blanco, Francesco Dal Santo, Teresa Bobes-Bascarán, Clara Martínez-Cao, Ainoa García-Fernández, Pilar A. Sáiz, Julio Bobes

**Affiliations:** ^1^Servicio de Salud del Principado de Asturias (SESPA), Oviedo, Spain; ^2^Department of Psychiatry, Universidad de Oviedo, Oviedo, Spain; ^3^Centro de Investigación Biomédica en Red de Salud Mental (CIBERSAM), Madrid, Spain; ^4^Instituto de Investigación Sanitaria del Principado de Asturias (ISPA), Oviedo, Spain; ^5^Instituto Universitario de Neurociencias del Principado de Asturias (INEUROPA), Oviedo, Spain; ^6^Department of Psychology, Universidad de Oviedo, Oviedo, Spain

**Keywords:** functioning, recovery, schizophrenia, monotherapy, antipsychotic

## Abstract

**Introduction:** Interest in the idea of recovery for certain patients with schizophrenia has been growing over the last decade. Improving symptomatology and functioning is crucial for achieving this. Our study aims to identify those factors that substantially contribute to real-world functioning in these patients.

**Methods:** We carried out a cross-sectional study in stable outpatients with schizophrenia on maintenance antipsychotic monotherapy. *Patients*: We studied 144 outpatients with schizophrenia (DSM-IV-TR criteria) meeting the following criteria: (1) 18–65 years of age; (2) being clinically stable for at least the previous three months; (3) on maintenance antipsychotic monotherapy (prescriptions ≤ 10 mg olanzapine, ≤200 mg quetiapine, or ≤100 mg levomepromazine as hypnotics were also allowed); and (4) written informed consent. *Assessment*: We collected information on demographic and clinical variables by using an *ad hoc* questionnaire. For psychopathology, we employed the Spanish versions of the following psychometric instruments: the Positive and Negative Syndrome Scale (PANSS), the Brief Negative Symptom Scale (BNSS-Sp), and the Calgary Depression Scale (CDS). In addition, cognitive domains were assessed using the Verbal Fluency Test (VFT), the Digit Symbol Substitution Test (DSST), and the Trail Making Test, parts A and B (TMT-A and TMT-B). Finally, we employed the Spanish versions of the University of California San Diego Performance-based Skills Assessment (Sp-UPSA) and the Personal and Social Performance (PSP) for assessing functional capacity and real-world functioning, respectively. *Statistical analysis*: A forward stepwise regression was conducted by entering those variables significantly associated with PSP total score into the univariate analyses (Student's *t*-test, ANOVA with Duncan's *post-hoc* test, or bivariate Pearson correlation).

**Results:** A total of 144 patients; mean age 40 years, 64% males, mean length of illness 12.4 years, PSP total score 54.3. The final model was a significant predictor of real-world functioning [*F*_(7, 131)_ = 36.371, *p* < 0.001] and explained 66.0% of the variance. Variables retained in the model: BNSS-Sp abulia, asociality, and blunted affect, PANSS general psychopathology, Sp-UPSA transportation, TMT-B, and heart rate.

**Conclusion:** Our model will contribute to a more efficient and personalized daily clinical practice by assigning specific interventions to each patient based on specific impaired factors in order to improve functioning.

## Introduction

Although schizophrenia has traditionally been linked to severe functional deficits, interest in the idea of recovery as a possibility for particular patients has been growing in recent years. This concept of recovery is relatively new and still under construction in schizophrenia. It is complex, encompasses multiple aspects of the patient's life, and can be operationalized in two domains that are distinct, but to some extent interdependent, i.e., the objective or clinical and the subjective or personal (1–3).

The objective clinical recovery domain includes symptomatology and functioning, and it is well known that there is no direct relationship between the two (4). Furthermore, numerous studies have focused on functioning predictors, but their results are rarely replicated. Among the most consistently reported predictors are negative symptoms and cognitive performance (2, 5–15). Recently, a systematic review and evidence synthesis on the relationship between functional capacity, as measured by the University of California San Diego Performance-based Skills Assessment (UPSA), and different measurements of real-world functioning reported a connection between these two constructs that merits further investigation (16). In this sense, Menendez-Miranda et al. (9) quantified the relationship between the UPSA and the Personal and Social Performance scale (PSP), one of the most widely used real-world functioning measures, reporting that each one explained 17% of the variance of the other. Depressive symptoms have also been associated with worse functioning (10, 17), although the strength of this relationship was very weak (17). Gonzalez-Blanco et al. (15) found IL-2, a peripheral inflammatory biomarker, to be associated with worse functioning and negative and general psychopathology symptoms. Other predictors of functional remission or recovery in patients with schizophrenia identified by Vita and Barlati (3) in their review were education level, employment status, family economic status, age at onset, hospitalizations, relapses, adherence to treatment, and disorganization symptoms. Except for age at onset, all of these were reported by a single study.

Concerning treatment, Tandon et al. (18) concluded that side effects associated with second-generation antipsychotics substantially impact patient functioning and quality of life. However, they acknowledged that this is a convoluted, under-researched area. To better understand the relationship between different treatment options and outcomes measures, we need studies in naïve patients, but the difficulty enrolling them severely hampers the research (19).

With the above in mind, it can be hypothesized that different factors would have a small but significant effect on the real-world functioning of patients with schizophrenia. Therefore, identifying this plethora of factors would help to manage this disorder with greater precision and reduce its associated functional impairments through specific interventions aimed at their modification. Thus, this study aims to model the relationship between the real-world functioning of patients with schizophrenia on maintenance antipsychotic monotherapy and an extensive range of independent variables. We hypothesize that, in addition to negative symptoms and cognitive performance, depressive symptoms, somatic comorbidities, and functional capacity will be found to substantially contribute to patient functioning.

## Methods

### Design

This is a naturalistic, cross-sectional study carried out in outpatients with schizophrenia consecutively seen at either of the two mental health centers participating in the study. Both centers are in Oviedo, Northern Spain, and each has a catchment area population of about 80,000 inhabitants.

The study was conducted according to the Declaration of Helsinki (20). The Clinical Research Ethics Committee of Hospital Universitario Central de Asturias in Oviedo approved the study protocol (Ref. 127/15), and written informed consent was obtained from all participants before enrolment.

### Patients

A total of 144 outpatients with schizophrenia (DSM-IV-TR criteria) were included in the study. Inclusion criteria were: (1) 18–65 years of age; (2) being clinically stable for at least the previous 3 months (without hospitalizations, symptom exacerbation, or any significant change in the type/dose of antipsychotic treatment); (3) on maintenance antipsychotic monotherapy (prescriptions ≤ 10 mg olanzapine, ≤ 200 mg quetiapine, or ≤ 100 mg levomepromazine as hypnotics were also allowed); and (4) written informed consent. Having an intellectual disability disorder or acquired brain injury was the sole exclusion criteria.

The sample was collected between July 2017 and December 2019. Therefore, we consecutively offered the study to the patients meeting all the inclusion criteria and none of the exclusion criteria at the clinic.

### Assessment

Information on sociodemographic and clinical characteristics, including weight, heart rate, and blood pressure, was collected using an *ad hoc* questionnaire. To evaluate different psychopathological domains, we used the Spanish versions of the following psychometric instruments: Positive and Negative Syndrome Scale (PANSS) (21), Brief Negative Symptoms Scale (BNSS-Sp) (22), and Calgary Depression Scale for Schizophrenia (CDSS) (23). In all cases, higher scores indicate greater severity of the symptomatology.

Cognition was evaluated using phonemic (F-A-S) and semantic (animals) Verbal Fluency (VF) tests (24, 25), Digit Symbol Substitution Test (DSST) (26), and Trail Making Test parts A and B (TMT-A and TMT-B) (27). These tests measure the following cognitive domains:

- VF tests: attention, memory, verbal fluency, and executive functions (28)
- DSST: motor speed, attention, and visuoperceptual functions, but probably also associative learning and working memory (29) and
- TMT: Part A measures psychomotor speed, attention, and spatial organization, while Part B measures attention switching, mental flexibility, and recall (30).

For VF and DSST, lower scores indicate lower cognitive performance, while for TMT-A and -B, higher scores reflect lower cognitive performance.

We employed the Spanish version of the University of California San Diego Performance-based Skills Assessment (Sp-UPSA) to measure functional capacity (31). It assesses everyday adaptive skills, including finance management, communication, planning recreational activities, and transportation, under optimal conditions. The Sp-UPSA provides four domain scores (from 0 to 25 points) and a total score, potentially ranging from 0 to 100 points. In all cases, higher scores indicate better functional capacity.

Finally, real-world functioning was assessed using the Personal and Social Performance scale (PSP) (32). The PSP is a clinician-rated instrument that evaluates patient functioning in four areas of their lives: self-care, socially useful activities including work and study, personal and social relationships, and disturbing and aggressive behaviors. It provides scores in each of the four areas ranging from 0 to 6, where higher scores indicate worse functioning. It also provides a single total score ranging from 0 to 100, where higher scores reflect better personal and social functioning.

### Statistical Analysis

To estimate the proportion of patients with a PSP total score ≥70, starting from a population size of 250 patients on maintenance antipsychotic monotherapy at our clinics, a sample of 144 subjects is required. This sample size was determined for a 95% confidence interval, with a precision of ±5%, and taking into account that the true estimated proportion will be 33%.

The statistical analysis was performed using IBM SPSS Statistics for Windows, Version 24.0. For all tests, the significance level was set at *p* < 0.05. To explore the potential relationships between PSP total score and sociodemographic, clinical, and psychometric variables, we used Student's *t*-test, ANOVA with Duncan's *post-hoc* test, or bivariate Pearson correlation. Finally, to model the relationship between the PSP total score and all variables found to be significantly associated with it in the univariate analysis, we performed a multiple linear regression (forward stepwise regression). To avoid collinearity, for those psychometric instruments that provide subscale scores (i.e., PANSS total score, UPSA total score), we do not include the total score in the analysis, nor do we include redundant measures (i.e., PANSS negative subscale or Marder negative factor).

## Results

### Sociodemographic and Clinical Characteristics

Patient mean age was 39.67 (11.93), 63.6% were males, and 58.3% had a secondary school degree. Most of the patients were never married (72.2%), were living with their family of origin (64.6%), and were not working (70.1%), and almost half were receiving a mental disability benefit (43.8%).

Their mean length of illness was 12.36 (10.91) years. Most had had at least one previous hospitalization (70.8%), and 19.4% had a history of suicide attempts. In addition, 45.8% had psychiatric comorbidities (substance use disorders, especially tobacco), and 63.9% had physical comorbidities (see Table 1).

**Table 1:** Sociodemographic and clinical characteristics of the sample.

**Sociodemographic**	***n* (%)**	**Clinical**	***n* (%)**
Age [mean (sd)]	39.67 (11.93)	Length of illness (years) [mean (sd)]	12.36 (10.91)
Sex, males	92 (63.9)	Hospitalizations, yes	102 (70.8)
Marital status		No. of hospitalizations [mean (sd)]	2.04 (1.64)
Never married	104 (72.2)	Age at first hospitalization [mean (sd)]	28.15 (8.82)
Married^$^	40 (27.8)	Suicide attempts, yes	28 (19.4)
Living arrangement		No. of suicide attempts [mean (sd)]	1.82 (3.59)
Alone	16 (11.1)	Psychiatric comorbidities, yes	66 (45.8)
Family of origin	93 (64.6)	One disorder	57 (39.6)
Own family	30 (20.8)	Two disorders	7 (4.9)
Institutionalized	2 (1.4)	Three disorders	2 (1.4)
Other	3 (2.1)	SUD tobacco	65 (45.1)
Educational level		SUD cannabis	9 (6.3)
Primary school	38 (26.4)	SUD alcohol	3 (2.1)
Secondary school	84 (58.3)	SUD cocaine	1 (0.7)
University	22 (15.3)	Somatic comorbidities, yes	92 (63.9)
Years of education [mean (sd)]	14.09 (4.42)	One disease	47 (32.6)
Work status		Two diseases	28 (19.4)
Working	18 (12.5)	Three diseases	13 (9.0)
Not working^*^	101 (70.1)	Four diseases	2 (1.4)
Homemaker or student	25 (17.4)	Five diseases	2 (1.4)
Mental disability benefit, yes	63 (43.8)	Ophthalmological problems	45 (31.3)
Anthropometry and vital signs		Hypercholesterolemia	15 (10.4)
BMI [mean (sd)]	27.69 (5.28)	Hypertension	13 (9.0)
Low weight (<18.5)	3 (2.1)	Hypertriglycerides	10 (6.9)
Normal weight (18.5–24.9)	46 (31.9)	Constipation	8 (5.6)
Overweight (25–29.9)	50 (34.7)	Diabetes II	8 (5.6)
Obesity (≥30)	43 (29.9)	Respiratory disease	8 (5.6)
SBP [mean (sd)]	117.68 (14.68)	Hearing impairment	5 (3.5)
DBP [mean (sd)]	78.34 (10.74)	Migraine	5 (3.5)
Heart rate [mean (sd)]	80.79 (14.55)	Cardiovascular disease	3 (2.1)
		Hyperthyroidism	3 (2.1)

*sd, standard deviation;*

$ *Married includes married, living as married, widow, and divorced*.

* *Not working includes permanently disabled due to health conditions other than a mental disorder, temporarily disabled, retired, and unemployed. AP, abdominal perimeter; DBP, diastolic blood pressure; SBP: systolic blood pressure; SUD: substance use disorder*.

Concerning psychopharmacotherapy, all patients were on maintenance antipsychotic monotherapy (paliperidone 46.5%, aripiprazole 19.5%, olanzapine 13.9%, clozapine 4.9%, and only 3.5% of patients received typical antipsychotics). Almost half were on long-acting intramuscular antipsychotics (paliperidone LAI-3 months 20.1%, paliperidone LAI-1 month 13.9%, aripiprazole LAI 1-month 6.3%, fluphenazine decanoate 2.1%, and Risperdal Consta LAI 1.4%). Furthermore, 15% of patients were taking low doses of olanzapine (7.6%) or quetiapine (7.6%) as hypnotics, 19.4% were taking antidepressants and 43.8% at least one benzodiazepine (see Table 2).

**Table 2:** Prescribed pharmacological treatment in descending order of frequency.

**Monotherapy antipsychotic**	***n* (%)**	**Antidepressant**	***n* (%)**
Paliperidone LAI-3 months	29 (20.1)	None	116 (80.6)
mg [mean (sd)]/DE-OLZ1 mg	456.38 (142.4)/7.04	Escitalopram	11 (7.6)
Paliperidone LAI-1 month	20 (13.9)	Sertraline	6 (4.2)
mg [mean (sd)]/DE-OLZ1 mg	121.25 (48.8)/16.84	Desvenlafaxine	3 (2.1)
Olanzapine	20 (13.9)	Mirtazapine	2 (1.4)
mg [mean (sd)]/DE-OLZ1 mg	10.25 (8.6)/10.25	Venlafaxine	2 (1.4)
Aripiprazole oral	19 (13.2)	Bupropion	1 (0.7)
mg [mean (sd)]/DE-OLZ1 mg	11.71 (7.1)/8.30	Clomipramine	1 (0.7)
Paliperidone oral	18 (12.5)	Maprotiline	1 (0.7)
mg [mean (sd)]/DE-OLZ1 mg	5.66 (3.1)/14.18	Vortioxetine	1 (0.7)
Risperidone	10 (6.9)	**Benzodiazepines**	***n*** **(%)**
mg [mean (sd)]/DE-OLZ1 mg	3.2 (1.8)/8.42	None	81 (56.3)
Aripiprazole LAI-1 month	9 (6.3)	One	58 (40.3)
mg [mean (sd)]/DE-OLZ1 mg	255.56 (88.2)/8.97	Two	5 (3.5)
Clozapine	7 (4.9)	Lorazepam	29 (21.2)
mg [mean (sd)]/DE-OLZ1 mg	264.29 (143.5)/8.63	Clonazepam	10 (7.0)
Amisulpride	3 (2.1)	Clorazepate dipotassium	9 (6.3)
mg [mean (sd)]/DE-OLZ1 mg	233.33 (152.7)/6.09	Diazepam	7 (4.9)
Fluphenazine decanoate	3 (2.1)	Bromazepam	2 (1.4)
mg [mean (sd)]/DE-OLZ1 mg	29.17 (7.2)/58.34	Ketazolam	2 (1.4)
Quetiapine	2 (1.4)	Alprazolam	1 (0.7)
mg [mean (sd)]/DE-OLZ1 mg	712.50 (689.4)/22.08	Flurazepam	1 (0.7)
Risperdal consta LAI	2 (1.4)	Lormetazepam	5 (3.5)
mg [mean (sd)]/DE-OLZ1 mg	150.00 (70.7)/89.29	Zoplicone	2 (1.4)
Haloperidol	1 (0.7)	**Other treatments**	***n*** **(%)**
mg [mean (sd)]/DE-OLZ1 mg	60.00 (0.0)/81.08	None	118 (82.0)
Ziprasidone	1 (0.7)	Biperiden	19 (13.2)
mg [mean (sd)]/DE-OLZ1 mg	120.00 (0.0)/15.15	Gabapentin	4 (2.8)
**Antipsychotic as hypnotic**	***n*** **(%)**	Folic acid	1 (0.7)
None	121 (84.0)	Oxcarbazepine	1 (0.7)
Olanzapine	11 (7.6)		
mg [mean (sd)]	9.55 (1.5)		
Quetiapine	11 (7.6)		
mg [mean (sd)]	122.50 (58.3)		
Levomepromazine	1 (0.7)		
mg [mean (sd)]	25.00 (0.0)		

*DE-OLZ1 mg, Dose equivalent to Olanzapine 1 mg/day; LAI, long-acting injectable; mg, milligrams; sd, standard deviation*.

On the whole, patients included in the study had predominantly negative symptoms [PANSS Negative = 18.68 (5.57), PANSS Positive = 12.31 (5.30)], almost no depressive symptomatology [CDSS = 2.96 (3.62)] and performed relatively poorly on the cognitive tests [Verbal Fluency phonemic total = 26.03 (10.18), semantic animals = 16.70 (5.33), Digit Symbol Substitution = 41.73 (17.02), and TMT-A = 50.02 (25.89) and -B = 124.96 (67.19)]. Their functional capacity was mildly impaired [Sp-UPSA total score = 70.90 (14.56)], with communication being the domain most impaired [15.87 (4.75)] while transportation was the least [18.87 (4.72); see Table 1].

### Real-World Functioning and Its Predictors

Patients showed manifest impairment in real-world functioning [PSP total score = 54.33 (18.43)], and only 18.1% of the patients showed a reasonable level of functioning (PSP total score > 70). Useful activities and relationships were the areas most impaired [2.32 (1.29) and 2.31 (1.29), respectively].

As shown in Tables 3, 4, several independent variables were significantly associated with patient real-world functioning. All variables involving negative symptoms showed the strongest correlations, followed by general psychopathology and functional capacity. On the contrary, the cognitive domains showed the weakest correlation with functioning, with attention switching, mental flexibility, and recall being the most strongly related. Also, being female, having some higher education, working or a homemaker/student, not receiving a mental disability benefit, having no history of suicide attempts, and no alcohol or benzodiazepine use were associated with better real-world functioning.

**Table 3:** Statistically significant associations between PSP total score and independent (categorical) variables.

**Variables**	**Categories**	**PSP total score**	**Statistical test, *p***
		**Mean (S.D.)**	
Sex	Males	50.73 (18.22)	−3.217^a^, 0.002
	Females	60.69 (17.19)	
Education level	Primary school	51.16 (17.15)	4.328^b^, 0.015 Higher ≠ (primary = secondary school)^c^
	Secondary school	53.08 (18.61)	
	Higher	64.55 (17.07)	
Work status	Working	66.39 (14.76)	10.988^b^, <0.001 Not working ≠ (working = homemaker or student)^c^
	Not working^@^	49.96 (18.15)	
	Homemaker or student	63.28 (14.95)	
Mental disability benefit	No	58.53 (18.04)	3.203^a^, 0.002
	Yes	48.92 (17.62)	
History of suicide attempts	No	56.30 (17.69)	2.674^a^, 0.008
	Yes	46.14 (19.49)	
Alcohol use	No	51.08 (18.75)	−2.585^a^, 0.011
	Yes	59.00 (17.05)	
Benzodiazepine use	No	59.15 (18.06)	3.607^a^, <0.001
	Yes	48.46 (17.26)	

a *Student's t-test*.

b *ANOVA*.

c *Duncan's post-hoc test*.

*S.D., standard deviation*.

@ *Not working includes permanently disabled due to a health condition other than a mental disorder, temporarily disabled, retired, and unemployed*.

**Table 4:** Statistically significant associations between PSP total score and independent (continuous) variables.

**Variables**	Statistical test^**#**^, p^*^
Years of education	0.253^4^^**^
Length of illness (months)	−0.190^4^^*^
Weight (kg)	−0.195^4^^*^
Heart rate	−0.303^4^^**^
PANSS positive	−0.367^4^^**^
PANSS negative	−0.683^4^^**^
PANSS marder negative factor	−0.728^4^^**^
PANSS general psychopathology	−0.567^4^^**^
BNSS-Sp anhedonia	−0.648^4^^**^
BNSS-Sp distress	−0.428^4^^**^
BNSS-Sp asociality	−0.653^4^^**^
BNSS-Sp abulia	−0.705^4^^**^
BNSS-Sp blunted affect	−0.602^4^^**^
BNSS-Sp alogia	−0.375^4^^**^
CDSS	−0.347^4^^**^
VF Semantic (animals)	0.194^4^^*^
DSST	0.215^4^^**^
TMT-A	−0.290^4^^**^
TMT-B	−0.254^4^^**^
Sp-UPSA finance management	0.407^**^
Sp-UPSA communication	0.425^**^
Sp-UPSA planning recreational activities	0.296^4^^**^
Sp-UPSA transportation	0.463^4^^**^

# *Bivariate Pearson correlation*.

* *p = 0.05*

** *p = 0.01*.

*BNSS-Sp, brief negative symptoms scale, Spanish version; CDSS, calgary depression scale for schizophrenia; DSST, digit symbol substitution test; Kg, kilograms; PANSS, positive and negative syndrome scale; PSP, personal and social performance scale; TMT-A, trail making test, part A; TMT-B, trail making test, part B; Sp-UPSA, University of California San Diego performance-based skills assessment, Spanish version; VF, verbal fluency*.

Results of the multiple linear regression are shown in Table 5. The final model explained 66.0% of the variance (*R*^2^ = 0.660, standard error of the estimate = 10.928), and the model was a significant predictor of real-world functioning [*F*_(7, 131)_ = 36.371, *p* < 0.001]. As shown in Table 2, the seven variables retained in the model contributed significantly to it. The final predictive model was:

$$\begin{array}{l} PSP\;total\;score=78.189+\left(-1.626\;*\;BNSS\;Abulia\right)\\ \;+\;\left(-0.391\;*\;PANSS\;General\;Psychopathology\right)\\ \;+\;\left(-1.448\;*\;BNSS\;Asociality\right)\\ \;+\;\left(0.968\;*\;UPSA\;Transportation\;\right)\\ \;+\;\left(-846\;*\;BNSS\;Blunted\;Affect\right)\\ \;+\;\left(0.041\;*\;TMT-B\right)+\left(-0.161\;Heart\;Rate\right) \end{array}$$

Abulia and asociality were the independent variables that had the highest multicollinearity with other variables, but their variance inflation factor (VIF) values were very far from 5 (2.410 and 2.166, respectively, see Table 2).

**Table 5:** Multiple linear regression model predicting real-world functioning (PSP total score).

**Variables**	**B**	**S.E**.	**Beta**	***t***	***P***	**VIF**
Constant	78.189	8.766		8.919	<0.001	
BNSS-Sp Abulia	−1.626	0.493	−0.261	−3.296	0.001	2.410
PANSS general psychopathology	−0.391	0.145	−0.172	−2.698	0.008	1.571
BNSS-Sp asociality	−1.448	0.527	−0.206	−2.746	0.007	2.166
Sp-UPSA transportation	0.968	0.232	0.251	4.170	<0.001	1.394
BNSS-Sp blunted affect	−0.846	0.267	−0.217	−3.172	0.002	1.808
TMT-B	0.041	0.017	0.144	2.442	0.016	1.349
Heart rate	−0.161	0.068	−0.128	−2.379	0.019	1.123

*S.E., standard error; VIF, variable inflation factors. BNSS-Sp, brief negative symptoms scale, Spanish version; PANSS, positive and negative syndrome scale; PSP, personal and social performance scale; TMT-B, trail making test, part B; Sp-UPSA, University of California San Diego performance-based skills assessment, Spanish version*.

## Discussion

In line with our objective, we were able to model the relationship between the real-world functioning of patients with schizophrenia on maintenance antipsychotic monotherapy and several independent variables. Our hypothesis identified seven factors with a small but significant effect on patient real-world functioning. Negative symptomatology (BNSS abulia, asociality, and blunted affect subscales), general psychopathology (PANSS subscale), functional capacity (UPSA transportation domain), cognitive performance (Trail Making Test, part B), and heart rate were found to significantly contribute to predicting real-world functioning.

Unsurprisingly, negative symptomatology was found to be the most robust predictor of real-world functioning, as reported by other authors (8, 9, 11–15, 33). It is worth highlighting the advantage of using the BNSS over the PANSS negative subscale or the Marder negative factor. It allowed us to determine the relationships between these two domains more accurately. We found that three out of the five dimensions that constitute the negative syndrome (34)—abulia, asociality, and blunted affect—were retained in the model. In this respect, we previously found identical results in patients during their first 10 years of the disorder (mean age 31.7, mean length of illness 4.6 years) (15), although other authors (11, 13, 35) did not find any relationship between the so-called expressive dimension of the negative symptomatology (blunted affect and alogia) and functioning. However, as early as 2000; Jablensky et al. (36) had linked the deficit in language and communication with difficulties in daily life, interpersonal relationships, and social functioning. We find our result regarding the relevance of affective expression to real-world functioning of great interest since non-verbal language constitutes more than 90% of the oral communication between human beings, and communication is a crucial ability for appropriate functioning in our society. Thus, psychosocial interventions intended to improve language and communication difficulties in these patients could also help enhance their psychosocial functioning.

Although Szabo et al. (16), in their systematic review and evidence synthesis, did not find a significant impact of functional capacity, measured using the UPSA, on different measurements of real-world functioning [Specific Level of Functioning (SLOF), Global Assessment of Functioning (GAF), and Multidimensional Scale of Independent Functioning (MSIF)] some studies have demonstrated the opposite. For example, Menendez-Miranda et al. (9) and Galderisi et al. (11) reported a positive and significant influence of functional capacity on real-world functioning (PSP and SLOF, respectively), as we did in this study. Specifically, we found that out of the four domains included in the functional capacity construct, the use of public transportation was retained in the model. Like the other tasks included in the UPSA, this specific task reflects general abilities essential to independent living, such as planning and organizing (37), and would mediate the potential functional impact of cognitive performance (11, 38).

The impact of cognitive performance on functioning was also demonstrated in patients with schizophrenia (5–7, 11), but the majority of the studies proving their relationship were carried out more than 10 years ago. Recently, Galderisi et al. (11) reported an indirect neurocognitive effect on patient work skills and interpersonal relationships. Social cognition was the mediator in both areas of real-world functioning, while functional capacity acted as a mediator only for work skills. However, Gonzalez-Blanco et al. (15) did not find any effect. These contradictory results could be explained by the fact that in the first study, the performance in the different neurocognitive areas was considered a single factor, while in the second, each cognitive domain was considered separately. Our study found that attention switching, mental flexibility, and recall were the only cognitive domains retained in the model. But, to further cloud the issue they were associated with functioning in the unexpected direction (partial correlation coefficient = 0.041); that is, if the rest of the variables in the equation remain constant, an increase of 1 second in performing the TMT-B will result in an increase of 0.041 points in the PSP total score. The magnitude of the coefficient is indeed minimal, but its contribution to the model is significant. This unexpected result could have been due to multicollinearity between TMT-B and other independent variables in the model, but this is not the case, as shown in Table 2. The TMT-B is a multifactorial test that measures processing speed, complex attention, cognitive flexibility, inhibitory control, and for some authors working memory as well. It is known that it is influenced by several factors such as age, motor speed (related to the former), and years of education, among others. Although neither age nor educational level was included in the model, both significantly affected the results of the univariate analysis in the expected direction (results not shown).

Furthermore, some prospective studies reported the effect of cognition on functioning to be mediated by negative and general symptomatology (39, 40), and Gonzalez-Blanco et al. (15) also found a significant effect of general psychopathology on PSP total score, as we did. Thus, further research is required to elucidate the real contribution of the TMT-B on real-world functioning. Unfortunately, there are no TMT-B normative data for Spanish patients with schizophrenia, and our sample size does not allow us to conduct the analysis stratifying for these factors.

Increased heart rate has been described in patients with schizophrenia since Kraepelin and also demonstrated in untreated first-episode patients and healthy first-degree relatives (41). This autonomic dysfunction has been related to the excess cardiovascular and metabolic morbidity and mortality seen in these patients (41, 42), as well as those with positive symptomatology (43, 44) and impairment in psychosocial functioning (45), even in first-episode patients (46). Our results add more support to the negative impact of autonomic dysfunction on real-world functioning, providing a specific line of intervention to improve it. This relationship may be mediated by the cognitive impairment often present in these patients. In this sense, a recent meta-analysis confirmed a significant association between the presence of several cardiovascular risk factors and cognitive impairment in patients with schizophrenia (47); thus, cognitive impairment is a plausible mediating factor between autonomic dysfunction and poor real-world functioning.

Contrary to our hypothesis, our model did not include depressive symptoms. However, in the bivariate analysis, total score on the CDSS was significantly related to PSP total score. Therefore, we think our result is mainly connected to the sample characteristics and their effect on the statistical analyses. On the one hand, our patients had almost no depressive symptomatology when they were evaluated. On the other, negative symptomatology, which showed the most robust relationship to patient functioning, displaced depressive symptoms in the multivariate analysis.

It is beyond the scope of this study to detect differences in the level of functioning according to the pharmacological treatment patients were prescribed. Still, we would like to highlight that only benzodiazepine use was related to real-world functioning in the univariate analysis. Although this is highly speculative, the lack of relationship to the antipsychotic treatment could be associated with the inclusion criterion of a monotherapy regimen.

The main limitation of our study is its cross-sectional design. Long-term longitudinal studies could describe changes in real-world functioning and search for predictor variables of these changes. It would also be of great interest to conduct this research in first-episode schizophrenia patients before starting pharmacological treatment. Thus, we could more accurately delineate the influence of the different factors from the beginning of the disorder and design phase-specific interventions to modify their impact. As for our study's strengths, the extensive range of variables and domains included and the use of the BNSS to assess the negative syndrome of schizophrenia instead of the PANSS negative subscale are the two main ones. Finally, the condition of being on antipsychotic monotherapy is a novelty in this field.

In conclusion, we have provided clinicians with a simple formula that identifies the factors most substantially associated with patient real-world functioning. This formula will contribute to a more efficient and personalized daily clinical practice by assigning specific interventions to each patient based on specific impaired factors to improve functioning.

## Data Availability Statement

The raw data supporting the conclusions of this article will be made available by the authors, without undue reservation.

## Ethics Statement

The studies involving human participants were reviewed and approved by Clinical Research Ethics Committee of Hospital Universitario Central de Asturias in Oviedo (Ref. 127/15). The patients/participants provided their written informed consent to participate in this study.

## Author Contributions

MG-P, PS, and JB designed the study and wrote the protocol. LG-Á, LG-B, and FD managed the literature searches and analyses. LG-Á undertook the statistical analysis. MG-P wrote the first draft of the manuscript. All authors contributed to and have approved the final manuscript.

## Conflict of Interest

MG-P has been a consultant to and/or has received honoraria/grants from Angelini, Alianza OtsukaLundbeck, Instituto de Salud Carlos III, Janssen-Cilag, Lundbeck, Otsuka, and Pfizer. LG-Á has received honoraria from the 7th Framework Program European Union. LG-B has received honoraria/grants from the Spanish Foundation of Psychiatry and Mental Health, European Psychiatric Association, Otsuka, Lundbeck, Angelini, Janssen-Cilag and Pfizer. FS has received grants from the Spanish Foundation of Psychiatry and Mental Health. PS has been a consultant to and/or has received honoraria or grants from Adamed, CIBERSAM, European Comission, GlaxoSmithKline, Instituto de Salud Carlos III, Janssen-Cilag, Lundbeck, Otsuka, Pfizer, Plan Nacional Sobre Drogas and Servier. JB has received research grants and served as consultant, advisor, or speaker within the last 5 years for: AB-Biotics, Acadia Pharmaceuticals, Angelini, Casen Recordati, D&A Pharma, Exeltis, Gilead, GSK, Ferrer, Indivior, Janssen-Cilag, Lundbeck, Mundipharma, Otsuka, Pfizer, Reckitt-Benckiser, Roche, Sage Therapeutics, Servier, Shire, Schwabe Farma Ibérica, research funding from the Spanish Ministry of Economy and Competiveness–Centro de Investigación Biomedica en Red area de Salud Mental (CIBERSAM) and Instituto de Salud Carlos III-, Spanish Ministry of Health, Social Services and Equality—Plan Nacional sobre Drogas—and the 7th Framework Program of the European Union. The remaining authors declare that the research was conducted in the absence of any commercial or financial relationships that could be construed as a potential conflict of interest.

## Publisher's Note

All claims expressed in this article are solely those of the authors and do not necessarily represent those of their affiliated organizations, or those of the publisher, the editors and the reviewers. Any product that may be evaluated in this article, or claim that may be made by its manufacturer, is not guaranteed or endorsed by the publisher.
